# CD151 interacts with integrin beta 2 in B cell lymphomas

**DOI:** 10.1007/s00018-025-05747-0

**Published:** 2025-06-04

**Authors:** Philipp M. Hagemann, Angelique N. Kenyon, Alfredo Cabrera-Orefice, Abbey B. Arp, Eva A. M. Hesius, Michiel van den Brand, Sjoerd J. van Deventer, Daphne de Jong, Blanca Scheijen, Zijun Y. Xu-Monette, Ulrich Brandt, Cornelia G. Spruijt, Michiel Vermeulen, Martin ter Beest, Ken H. Young, Annemiek B. van Spriel

**Affiliations:** 1 Department of Medical BioSciences, Radboud Institute for Medical Innovation, Radboud University Medical Center, Nijmegen, The Netherlands; 2 Radboud Institute for Medical Innovation, Radboud University Medical Center, Nijmegen, The Netherlands; 3 Department of Hematology, Radboud University Medical Center, Nijmegen, The Netherlands; 4 Pathology-DNA, Rijnstate Hospital, Arnhem, The Netherlands; 5 Department of Pathology, Radboud University Medical Center, Nijmegen, The Netherlands; 6 Department of Pathology, Antoni Van Leeuwenhoek Hospital/The Netherlands Cancer Institute, Amsterdam, The Netherlands; 7 Hematopathology Division and Department of Pathology, Duke University Medical Center, Durham, NC USA; 8 Department of Molecular Biology, Faculty of Science, Oncode Institute, Radboud University Nijmegen, Nijmegen, The Netherlands; 9 Division of Molecular Genetics, The Netherlands Cancer Institute, Amsterdam, The Netherlands

**Keywords:** TSPAN24, CD18, Non-Hodgkin lymphoma, Membrane protein, Cell spreading

## Abstract

CD151 is a broadly expressed four-transmembrane protein (tetraspanin) that interacts with laminin‐binding integrins like integrin alpha 3 (ITGA3). CD151 drives tumor development and expression correlates with poor prognosis in solid cancers, but CD151 has not been studied in B cell malignancies. We investigated CD151 expression on normal human B cells and B cell lymphomas using highly sensitive flow cytometry and immunohistochemistry. Expression of CD151 increased during B cell differentiation from naïve to memory B cells to plasma cells. B lymphoma cell lines and human lymphoma biopsy samples expressed higher levels of CD151 compared to normal B cells, but CD151‐deficient lymphomas were identified as well. To investigate the function of CD151 in B cells, CD151‐deficient and stably transduced CD151 expressing B lymphoma cell lines were generated. Immunoprecipitation-mass spectrometry analysis of CD151 protein complexes identified integrin beta 2 (ITGB2) as new interaction partner in lymphoma cells. Deficiency of CD151 decreased cell surface expression of alpha integrin subunits L (ITGAL) and M (ITGAM), and impaired ICAM-1-mediated cell spreading. Interestingly, B cells and lymphomas did not express ITGA3‐bound CD151 compared to T cells that expressed two different populations of integrin‐bound and integrin‐free CD151. Despite CD151 expression not being related to clinical outcome of patients with diffuse large B cell lymphoma (DLBCL), CD151 expression was predominantly detected in the activated (ABC) subset of DLBCL. Taken together, we identified a new molecular association of CD151 with ITGB2, and targeting integrin-free CD151 in DLBCL may represent a new target for immunotherapy.

**Supplementary Information:**

The online version contains supplementary material available at 10.1007/s00018-025-05747-0.

## Introduction

CD151 is a four-transmembrane protein belonging to the superfamily of tetraspanins [1]. Tetraspanins play an important role in cancer development, progression, and metastasis [2]. They are palmitoylated small four-transmembrane proteins that bind in cis with other membrane proteins through their large extracellular loop (LEL) organizing them in tetraspanin nanodomains [3–7]. CD151 plays a critical role in cell motility and adhesion [8, 9]. It is broadly expressed in human tissues and can be found on different types of hematopoietic cells.

In humans, mouse and zebrafish models, CD151 deficiency has been implicated in kidney failure, bullous skin lesions, nail dystrophy, defective wound healing, and sensorineural deafness [10–14]. In addition, CD151 is a driver of cancer development, progression, and metastasis [15–17]. Increased expression of CD151 has been correlated with poor prognosis in patients with lung, endometrial, and pancreatic cancer, as well as esophageal squamous cell carcinoma [15, 18–26].

Different mechanisms have been described for how CD151 elicits its functions in driving cancer and metastasis. CD151 interacts with beta 1 integrins, growth factor receptors, and matrix metalloproteinases [19, 27]. Integrin alpha 3 (ITGA3) and integrin alpha 6 (ITGA6) were identified as direct binding partners of CD151 [28–33]. The binding sites were characterized to involve amino acid (aa) 570–705 in ITGA3 and aa 186–217 in the LEL of CD151 [34, 35]. Importantly, antibodies that bind CD151 in this epitope region block ITGA3 binding and inhibit metastasis [34, 36]. Based on these findings, two states of CD151 have been defined, namely CD151 associated with ITGA3 (CD151^bound^) or lacking association with ITGA3 (CD151^free^). Integrin-free CD151 has been reported to facilitate integrin-independent cell motility and correlates with tumor progression and metastasis [37]. Clustering of CD151^free^ with antibodies inhibited tumor cell motility in vivo [8] and inhibited metastasis by blocking de-adhesion of tumor cells [9]. Previous studies reported CD151 expression on activated T cells [38–41] which was related to proliferation of human and murine T cells [40, 42]. In B cells and B cell-derived cancers, CD151 expression has not been investigated.

In this study, we report the differential expression levels of CD151 within the human mature B cell compartment. In addition, we detected a significant increase in CD151 expression in diffuse large B cell lymphoma (DLBCL) and follicular lymphoma (FL) compared to healthy B cells. Although CD151 expression was not related to clinical outcome of DLBCL, CD151 expression was predominantly detected in the more aggressive activated B cell (ABC) subset of DLBCL. We identified integrin beta 2 (ITGB2, CD18) as a novel binding partner of CD151 in B cell lymphoma.

## Materials and methods

### Antibodies

All antibodies used in this study are listed in Supplementary Table 1.

### Human cell lines and primary cells

All cell lines were cultured in RPMI-1640 (Thermo Fisher Scientific #11875093) supplemented with 1% stable glutamine (Capricorn Scientific STA-B), 1% antibiotics/antimycotics (Thermo Fisher Scientific #15240062), 10% fetal bovine calf serum (FCS) and kept at 37 °C, 5% CO_2_, and 90% humidity. Cell lines used were BJAB (Burkitt lymphoma, Cat: ACC 757), Daudi (Burkitt lymphoma, CAT: ACC 78), OCI-Ly1 (GCB DLBCL, Cat: ACC 722), OCI-Ly8 (GCB DLBCL), Raji (Burkitt lymphoma. Cat: ACC 319), SU-DHL-2 (DLBCL, Cat: ACC 902), SU-DHL-5 (GCB DLBCL, Cat: ACC 571), SU-DHL-6 (GCB DLBCL, Cat: ACC 572), SU-DHL-10 (GCB DLBCL, Cat: ACC 576), TMD8 (ABC DLBCL), U-2932 (ABC DLBCL, Cat: ACC 633), WSU-NHL (GCB DLBCL, Cat: ACC 58). Cells lines were obtained from DMSZ (ACC) or from the Department of Pathology (Radboudumc) and validated using STR analysis. All cell lines were regularly tested for mycoplasma.

Primary cells were obtained from healthy individuals with informed consent in accordance with institutional and international guidelines following the Declaration of Helsinki. For peripheral blood lymphocytes (PBLs), buffy coats were supplied by Sanquin Nijmegen. Lymphocytes were purified via Ficoll density centrifugation (2100 RPM, 25 min, RT). Tonsillar lymphocytes were obtained from surgery (Department of Pathology of CWZ Hospital, Nijmegen, the Netherlands). Tonsils were cut into small pieces and incubated in cell culture medium in 50 mL tubes at 4 °C on a roller. Obtained cell suspension was passed through a 100 µm mesh and incubated as described for cell lines or frozen in 90% FCS, 10% DMSO for later use.

### Transfection of cell lines and generation of CD151-deficient B cells

Transfection of BJAB and Raji cells was performed with Neon™ Transfection System (Thermo Fisher Scientific MPK5000) with 1 pulse for 40 ms at 1350 V. Stable CD151-deficient cell lines were generated by CRISPR-mediated genome editing of the CD151 gene, surface staining for CD151 followed by sorting of CD151-deficient cells with a Melody cell sorter (BD Biosciences). For the generation of stable GFP-tagged CD151 expressing cell lines, BJAB cells were transfected as described above and sorted for GFP expression and CD151 surface expression four times. For immunoprecipitation of GFP-tagged CD151 in CD151-deficient BJAB cell line, 100*10^6 BJAB cells were suspended in 20 mL of supplied R buffer containing 50 µg/mL plasmid and cells were transfected with Neon™ Transfection System. Cells were suspended in antibiotic-free RPMI-1640 medium, containing 10% FCS and 1% stable glutamine and cultured overnight. For the generation of CD151-deficient cell lines, px330 (Addgene #42230) vector was used. Guide RNA sequences are provided in Table S2. Expression plasmids containing wildtype CD151 and N- and C-terminally tagged CD151 were purchased form GenScript (# NM_004357.5). ITGB2—mYFP was a gift from Timothy Springer (Addgene plasmid # 8638) [43]. Plasmids for differently tagged CD151 and ITGB2 were generated by using the Q5 Site-Directed Mutagenesis Kit (New England Biolabs) according to the manufacturer’s protocols and using custom primers. Constructs were verified by Sanger sequencing.

### Flow cytometry

Surface staining was performed by blocking non-specific binding of antibodies with 2% human serum in PBS containing 1% BSA and 0.01% sodium azide (PBA) for 10 min on ice. Cells were stained with primary labeled antibodies for 30 min on ice. After washing, cells were stained with live dead discrimination dye eFL780 (eBiosciences #65–0865-18), diluted 1 to 10,000 in PBS, for 10 min RT. After staining cells were washed twice in PBA, resuspended in PBA and measured the same day using the FACS Lyric or FACS Verse from BD. For some experiments, cells were fixed in PBS containing 2% formaldehyde for 10 min RT, washed in PBS, resuspended in PBA and measured within a week. Data was analyzed using FlowJo X Software (FlowJo LLC).

### Immunohistochemistry

CD151 expression on formalin-fixed paraffin-embedded (FFPE) tissue microarray (TMA) slides [44] was detected with 3,3′-Diaminobenzidine (DAB) staining. TMA slides were deparaffinized with xylene and ethanol. Slides were washed with water and antigen heat retrieval was performed with pH 6 citrate buffer for 10 min cooking in a microwave and 20 min incubation in hot buffer. Slides were washed with water and blocked with 5% BSA for 2 h RT. Slides were incubated with primary antibody against CD151 clone E4I9 J diluted in 1% BSA containing PBS and incubated overnight at 4 °C. On the following day, slides were washed with PBS containing 0.1% Tween 20 and blocked for peroxidases 10 min with EnVision Flex Peroxidase-blocking reagent (Darko #K8000). After blocking, slides were washed 5 times with PBS. Slides were incubated with secondary antibody HRP dextramer 1 h RT. After 4 times washing, slides were stained with DAB for 8 min and washed in water. Counterstain with haematoxylin was performed with 20 s incubation and 5 min incubation in water. Slides were mounted with Pertex Mounting Medium (Beldico #5500552). Digital scans of TMAs were scored for CD151 expression. Detailed information regarding the cohort, as well as calculations of overall survival (OS) and progression-free survival (PFS), can be found in [45].

### Immunoprecipitation, mass spectrometry, and western blot

Cell lysates of GFP-tagged CD151 expressing BJAB cells were prepared with 1% IGEPAL CA-630 in PBS with the addition of 1 tablet of cOmplete™ Protease Inhibitor Cocktail (Merck #4693116001) per 50 mL buffer on ice. GFP was pulled down with ChromoTek GFP-Trap® Agarose beads (ChromoTek). Beads were washed 3 times with PBS. Proteins were eluted by heating of beads to 95°C for 5 min in sample buffer (BioRad #1610747). Eluted IP samples were loaded onto 10% Tricine gels. Gels were run for 20 min at 50 V for a total migration distance of approximately 0.5 cm. Gel spots were cut, fixed in 50% methanol, 10% acetic acid, 10 mM ammonium bicarbonate, destained in 10% acetic acid and in-gel digested with trypsin. Resulting tryptic peptides were separated by liquid chromatography and analyzed by tandem mass spectrometry (LC–MS/MS) in a Q Exactive Orbitrap Mass Spectrometer equipped with an Easy nLC1000 instrument (Thermo Fisher Scientific). MS raw data files were analyzed using MaxQuant (v1.5.0.25) using default settings. The search was performed against the Homo sapiens reference proteome retrieved from UniProt (01.01.2023) including known protein contaminants. Individual protein abundances were determined by label-free quantification (LFQ) and intensity-based absolute quantification (iBAQ) values.

To confirm CD151-ITGB2 interaction, western blot was used. BJAB cells were transfected as described above with CD151-GFP and ALFA-tagged ITGB2. Transfected cells were harvested after one day of incubation and lysed as described above. IP was performed either by pulling on GFP or the ALFA tag with selector ST beads (NanoTag Biotechnologies). Eluted samples were loaded onto 4-15% SDS PAGE (BioRad #4561084) and transferred via tank blotting to 45 µm pore size PVDF membrane (Merck). Membrane was blocked with 5% BSA in PBS and stained for GFP-tag or ALFA-tag. Blots were visualized on a Typhoon (Cytiva).

For co-immunoprecipitation of GFP-CD151 with endogenous ITGB2, 10*10^6 cells were collected, washed with PBS and lysed in IP-lysis buffer containing 1% IGEPAL, 25 mM Tris–HCl pH 7.5, 150 mM NaCl, 5 mM MgCl_2_ supplemented with protease and phosphatase inhibitors (Merck) for 30 min at 4°C with end over end rotation. Samples were centrifuged for 15 min at 12,000 RPM and supernatants were transferred to a fresh tube. An aliquot was taken for the total lysate sample and 25 µL of washed GFP-Trap Magnetic Agarose (Chromotek) was added to the lysates and incubated for 2 h at 4°C with end over end rotation. After the incubation, samples were washed 4 times with IP-lysis buffer using magnetic separation. After the last wash, residual buffer was removed and samples were stored at −80°C until analysis by western blotting. Samples for western blotting were prepared by taking 5*10^6 cells, washing the cells 2 × with PBS and lysis in 200 µL of RIPA buffer (1% IGEPAL, 0.25% sodium deoxycholate, 50 mM Tris–HCl pH 7.5, 150 mM NaCl, 1 mM EDTA, 0.1% SDS, protease inhibitors, phosphatase inhibitors) for 30 min on ice. Insoluble material was removed by centrifugation for 15 min at 12,000 RPM. For equal loading, protein concentration of the samples was determined using a Micro BCA kit (Pierce). Samples were analyzed on 7.5% or 10% SDS-PAA gels. Samples were boiled for 5 min in SDS sample buffer with β-mercaptoethanol as a reducing agent. For western analysis, samples were transferred to 0.45 µm Immobilon-FL PVDF membrane (Millipore) and blocked with Milk blocking buffer (Pierce). GFP was detected using anti-GFP. For detection of integrin alpha L (ITGAL, CD11a), integrin alpha M (ITGAM, CD11b), integrin alpha X (ITGAX, CD11c) and CD151 blots were probed with goat anti rabbit-HRP and signal was obtained using the Clarity Max Western ECL Substrate (BioRad #1705062) and detected using the ImageQuant LAS-4000 Multi-Mode Imager (GE). For the other blots, donkey IRDye conjugated antibodies were used and imaged with a Typhoon Imager (Cytiva).

### Cell adhesion assay

A 10% (w/v) solution of BSA in PBS was heated to 70°C for 30 min and used as blocking agent (hBSA). 96 well plates were coated with 40 µL coating solution (freshly prepared 10 µg/mL anti-IgM in PBS, 1 µg/mL ICAM-1-Fc (low) in PBS, 10 µg/mL ICAM-1-Fc (high) in PBS, or 10% hBSA in PBS). Plates were incubated at 37°C for 1 h. Plates were washed 5 times with 300 µL PBS. Each well was incubated with 50 µL 10% hBSA PBS for 1 h at RT. Plates were washed 5 times with 300 µL PBS.

Cells were labeled with CFSE for later detection with a UV/Vis plate reader capable of measuring fluorescein. Cells were centrifuged and resuspended in 3 mL CFSE 1:3000 diluted in PBS. After incubation for 10 min at 37°C, 10 mL cell culture medium was added, and cells were centrifuged. Cells were washed twice and resuspended in adhesion medium (RPMI 1640, 2 mM L-Glu, 0.5% BSA, 1 mM CaCl_2_, 1 mM MgCl_2_, 50 µM β-mercaptoethanol) at a density of 0.5*10^6 cells/mL. 50 µL of the cell suspension was added to each well, resulting in 25,000 cells/well. The plate was centrifuged for 15 s (1500 RPM) to spin cells to the bottom of the plate. Cells are then immediately incubated at 37°C for 30 min. Afterwards, cells that did not adhere to the coated wells are washed away by addition of 300 µL PBS with Ca^2+^ and Mg^2+^ and decanting of the plate (performed 5-times). Cells were lysed by addition of 30 µL lysis buffer (0.1 triton x-100 in PBS with 0.1 N NaOH) and incubation at 37°C for 30 min. Fluorescence intensity of lysed cells was measured in a plate reader with optimal settings, here for fluorescein, Ex: 485/20, Em: 530/30.

### Confocal microscopy

Cleaned coverslips were coated with 0.1% w/v poly-L-lysine. Cells were seeded at a density of 0.5*10^6 cells/mL for 30 min at 37°C in IMDM medium containing 10% FCS. Cells were fixed with a 4% formaldehyde PBS solution at RT for 1 h. Blocking was performed with PBS containing 3% BSA, 10% horse serum, 10 mM glycine for 30 min at RT. Cells were permeabilized with 0.2% Tween 20 dissolved in PBS for 30 min RT. Staining was performed in 0.1% Tween 20 containing PBS with 3% BSA and 10% horse serum. Cells were stained for 1 h at RT with 10 µg/mL primary antibodies. After thorough washing, cells were incubated with 10 µg/mL secondary antibody and incubated for 30 min RT. Coverslips were washed and mounted on microscope slides with ProLong Glass Antifade Mountant (Invitrogen). Images were acquired using Zeiss LSM900 with Airyscan2, in confocal mode with a 63X NA 1.4 oil objective. Analysis and quantification were done in FIJI. Co-localization was performed after background correction with a threshold to remove unspecific background signals.

### Cell spreading assay

Cleaned coverslips were coated with freshly prepared 10 µg/mL ICAM-1-Fc diluted in PBS and were incubated for 1 h at 37°C followed by 3 washes with PBS. A 10% (w/v) solution of BSA in PBS was heated to 70°C for 30 min and used as blocking agent (hBSA). Blocking was performed for 1 h at RT, followed by 5 washes with PBS. Cells were washed once, and resuspended in adhesion medium at a density of 0.5*10^6 cells per 100 µL and allowed to adhere for 30 min at 37°C. Cells were fixed with a 4% formaldehyde PBS solution for 20 min at RT. Permeabilization was done with 0.5% saponin dissolved in PBS for 15 min at RT. Membrane staining was performed using phalloidin-488 in PBS with 0.5% saponin for 30 min at RT. After thorough washing with PBS, cells were stained for 5 min at RT with 0.3 μg/mL 4’−6-diamidino-2-phenylindole (DAPI) in PBS. Coverslips were washed in PBS and MilliQ and mounted on microscope slides with Fluoromount-G (SouthernBiotech). Images were acquired using a Zeiss LSM900 with Airyscan2, in Airyscan mode using a 63X NA 1.4 oil objective. Analysis and quantification were done in FIJI. A threshold was set on the cell via the cell/glass interface, followed by tracing the entire cell body which was added to the roi manager. Using the measurement tool the total cell area and perimeter were measured. The area represents the amount of space inside the cell boundary while the perimeter represents the total length of the cell boundary.

### Colocalization

Cleaned coverslips were coated with 0.1% w/v poly-L-lysine. Cells were washed once with PBS and seeded at a density of 0.5*10^6 cells per 100 µL for 30 min at 37°C in PBS. Cells were fixed with a 4% formaldehyde PBS solution for 20 min at RT. Blocking with simultaneous permeabilization was performed with 3% BSA, 10 mM glycine, 1% goat serum, 2% human serum, 0.5% saponin in PBS for 30 min at RT. Staining was performed in block/perm buffer with 10 µg/mL primary antibodies for 1 h at RT. After thorough washing, cells were incubated with 10 µg/mL secondary antibody and incubated for 30 min RT. Coverslips were washed with PBS, cells were stained for 5 min at RT with 0.3 μg/mL DAPI in PBS. Coverslips were washed with PBS and MilliQ and mounted on microscope slides with Fluoromount-G. Images were acquired using a Zeiss LSM900 with Airyscan2, in confocal mode with a 40X NA 1.0 water objective. Analysis and quantification were done in FIJI. Co-localization was performed using FIJI plugin BIOP-JACoP.

### Expression data analysis

Data of RNA expression was obtained from publicly available data from the DepMap project [46, 47] for expression analysis in cell lines and the German MMML consortium [48] for expression analysis in human lymphomas and analyzed with R.

### Statistical analysis

Statistics were performed with R and GraphPad Prism 10. Statistical significance was defined as *P* ≤ 0.05. N refers to the number of independent experiments, n to the number of samples. Specific statistics are included in figure legends.

## Results

### CD151 expression is detected in normal and malignant B cells and increases during B cell maturation

**Fig. 1:**
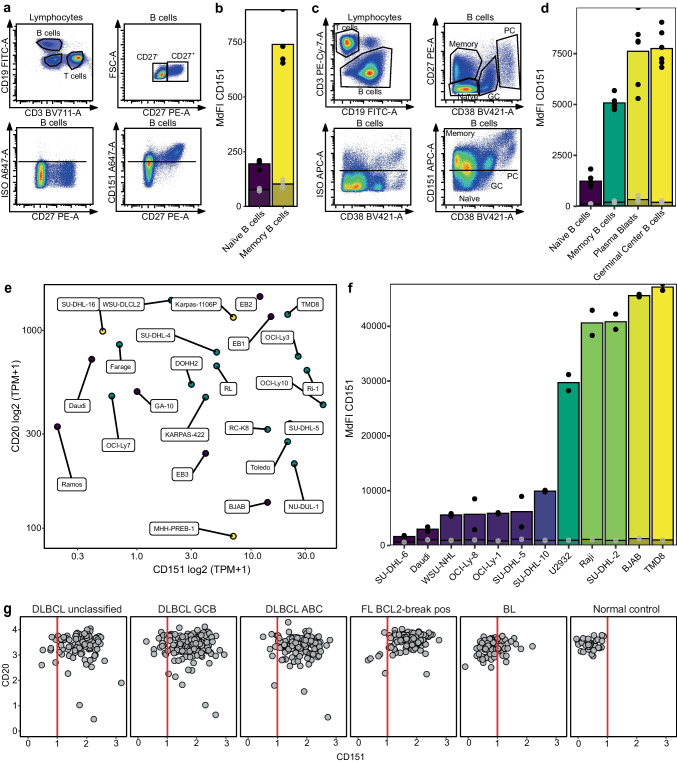
CD151 is expressed by human B cells and B cell lymphomas. **a** Protein expression of CD151 was investigated with surface staining of PBMCs with clone 11G5a and gated for naïve (CD3^−^, CD19^+^, CD27^−^) and memory B cells (CD3^−^, CD19^+^, CD27^+^) using flow cytometry. **b** Quantification of panel a: Median fluorescence intensity (MdFI) of CD151 plasma membrane expression on memory and naïve B cells from PBMCs ( N = 3). **c** Tonsillar lymphocyte were gated for naïve (CD3^−^, CD19^+^, CD27^−^, CD38^−^), memory (CD3^−^, CD19^+^, CD27^+^, CD38^−^), germinal center B cells (CD3^−^, CD19^+^, CD27^low^, CD38^+^), and plasma cells (CD3^−^, CD19^+^, CD27^high^, CD38^high^). **d** Quantification of panel c: MdFI of CD151 plasma membrane expression on tonsillar B cells ( N = 3). **e** mRNA expression of CD151 and CD20 in B cell lymphoma cell lines from the DepMap project. Cell lines are distinguished by color-coded disease classification: Burkitt lymphoma (dark blue), diffuse large B-cell lymphoma (green), other B cell lymphomas (yellow). **f** Expression of CD151 on selected B cell lymphoma cell lines stained with biotinylated CD151 antibody clone 11G5a (black points) compared to isotype control (grey points) ( N = 2). **g** Normalized mRNA expression of CD151 in different primary lymphomas (DLBCL, FL, BL) and normal B cells compared to CD20 expression. Red line indicates expression in normal control. Data from German MMML consortium (see method section)

Previous reports on CD151 as activation marker in T cells sparked our interest to study CD151 expression in the lymphoid compartment [40, 41]. In the past, using low resolution flow cytometry, lymphocytes and especially B cells have been reported to be mostly negative for CD151 expression [49]. However, staining human blood mononuclear cells (PBMCs) (Fig. 1a,b) and tonsillar lymphoid cells (Fig. 1c,d) resulted in clear detection of cell surface expression of CD151 on B cells. From peripheral blood and tonsils, naïve, memory, germinal center (GC) B cells and plasma blasts were analyzed for the presence of CD151. CD151 protein expression was low in naïve B cells, increased in memory B cells, and was highest in GC B cells and plasma blasts. To investigate CD151 expression in different B cell lymphoma cell lines, publicly available data from the DepMap project was used [46, 47]. Within the group of lymphoma cell lines, a range of CD151 mRNA expression was observed that was not linked to a specific type of B cell lymphoma (Fig. 1e). The relationship between mRNA levels and protein expression is not always linear, therefore CD151 protein expression was determined in B lymphoma cell lines using biotinylated monoclonal antibodies in high resolution flow cytometry experiments. Most lymphoma cell lines showed detectable CD151 cell surface protein expression, which varied in levels (Fig. 1f). While SU-DHL-6 and Daudi cells showed no CD151 expression, BJAB, Raji, and TMD8 cell lines expressed high levels of surface located CD151. We verified CD151 expression in primary human lymphomas (Fig. 1g) and analyzed publicly available data from the German MMML consortium (Molecular Mechanisms in Malignant Lymphoma) for CD151 mRNA expression [48]. CD151 expression was highest in DLBCL and FL, and lowest in Burkitt lymphoma (BL) and primary B cells (Fig. 1g). Together, these data show that CD151 expression is higher on lymphomas compared to normal B cells, where its expression increases during B cell differentiation.

### CD151 interacts with ITGB2

**Fig. 2:**
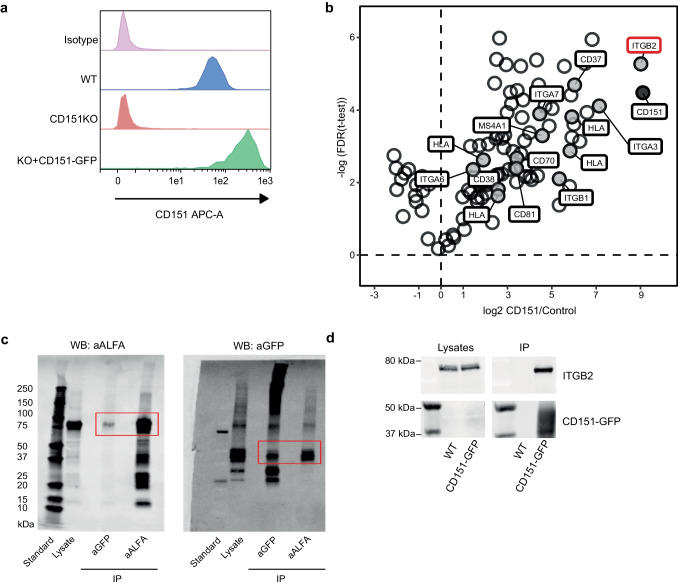
CD151 interacts with ITGB2. **a** Generation of BJAB cell lines deficient for CD151 (CD151KO) and over expressing GFP-tagged CD151. Histogram of surface stained CD151 (antibody clone 11G5a) using flow cytometry. **b** Volcano plot of proteome data. Enrichment of CD151 pulldown over control beads is shown with log2 scaling and the false discovery rate of performed t-test as -log 10. CD151 is marked dark grey, integrins and B cell marker proteins grey. N = 3, one representative experiment of three shown. Full list can be found in Suppl. Table 3. **c** Pulldown (IP) of CD151 and ITGB2 in lysates from BJAB cells transiently transfected with ALFA-tagged ITGB2 and GFP-tagged CD151. Western blots were stained for ALFA-tag and GFP to visualize ITGB2 (80 kDa) and CD151-GFP (50 kDa), respectively (N = 2). **d** Pulldown (IP) of GFP-tagged CD151 in lysates from BJAB cells (WT and CD151-GFP) showing CD151 interaction with endogenous ITGB2. Western blots were stained for ITGB2 (80 kDa) and CD151-GFP (50 kDa) (N = 3)

Next, we investigated which proteins can interact with CD151 in B lymphoma cells. We characterized proteins associated with CD151 using co-immunoprecipitation (co-IP) followed by protein identification with mass spectrometry. As a model, BJAB cells were engineered to be deficient for endogenous CD151 (CD151 KO) using CRISPR-Cas9 technology, and complemented by stably expressed GFP-tagged CD151 (Fig. 2a). CD151 protein expression in these cell lines was validated using flow cytometry (Fig. 2a) and high affinity anti-GFP nanobodies were used to pulldown CD151. This system allows for studying protein interactions mediated by the LEL of CD151, which is not disturbed by the GFP pulldown (SFig. 1).

Immunoprecipitation of CD151 in these BJAB cell lines was specific as shown by CD151 being one of the top hits in the CD151-positive cells compared to the control (Fig. 2b, Suppl. Table 3). Identification of known CD151 binding partners (ITGA3, ITGA6 and integrin beta 1 (ITGB1)) further validated our approach. Interestingly, we identified ITGB2 as the most highly enriched binding partner of CD151 (Fig. 2b). Other CD151-interacting proteins included other tetraspanins (CD37 and CD81) as well as B cell associated proteins CD20 (*MS4A1*), HLA and CD38 (Fig. 2b and Suppl. Table 3). The interaction of CD151 with ITGB2 was shown in three independent experiments. To further confirm the interaction, ALFA-tagged ITGB2 and GFP-tagged CD151 were transiently transfected in BJAB cells and co-IPs for both proteins were performed. Both CD151 and ITGB2 were able to pulldown each other in IGEPAL CA-630-lysed cells shown by western blotting (Fig. 2c), validating the association of CD151 with ITGB2. Further assessment was done using IP experiments in lysates from BJAB wild-type (WT) and BJAB CD151-GFP expressing endogenous levels of ITGB2. Pulldown of GFP-tagged CD151 resulted in co-IP of endogenous ITGB2 (Fig. 2d, SFig. 2 for original blots). To assess if the interaction of CD151 and ITGB2 was affected by divalent cations that can regulate the activation status of integrins, IGEPAL lysis buffer supplemented with Ca^2+^/Mg^2+^, Mg^2+^, or EDTA was used. Pulldown (IP) of GFP-tagged CD151 showed that the interactions occur independently of the divalent cations, indicating that CD151 physically associates with endogenous ITGB2 in a constitutive fashion (SFig. 3).

### CD151 depletion affects expression of the alpha subunits of ITGB2

**Fig. 3:**
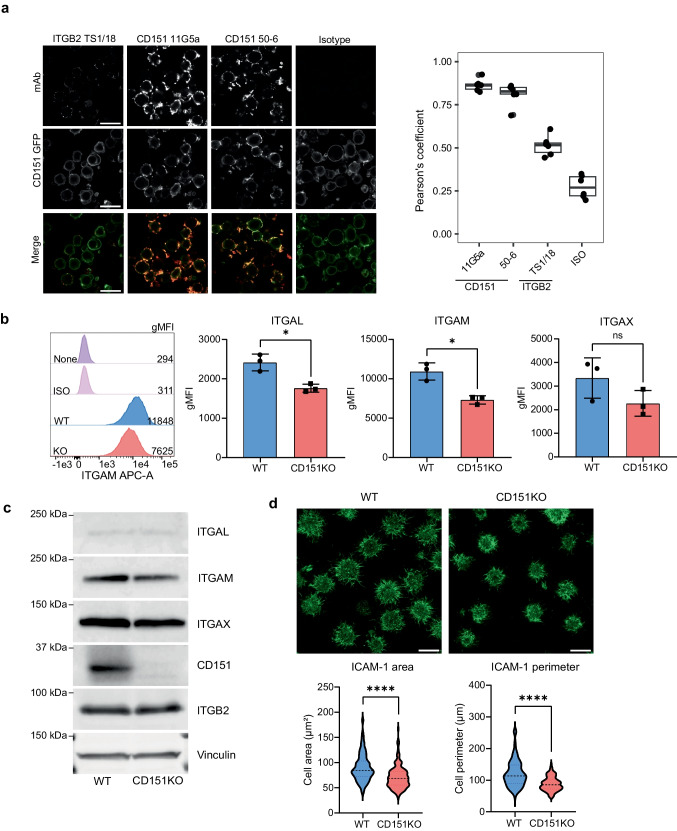
CD151 depletion affects expression of integrin alpha subunits and cell spreading. **a** Co-localization and correlation analysis of GFP-tagged CD151 with ITGB2, isotype (negative control) and CD151 (positive control). Representative images are shown, Pearson’s coefficients of the analyzed images are shown in the plot on the right. (Magnification 63X, scale bar = 10 µm) **b** Surface expression of ITGAL, ITGAM, and ITGAX was determined by flow cytometry on BJAB cell lines, one representative image is shown. Variation in surface expression between WT and CD151KO cells was determined by means of a two-tailed paired student’s t-test mean ± SD (*P < 0.05), shown on the right (N = 3). gMFI = geometric mean fluorescence intensity. **c** Western blot of alpha chains in lysates from BJAB WT and BJAB CD151KO cells. Blots were stained for ITGAL (180 kDa), ITGAM (165 kDa), ITGAX (145 kDa), CD151 (28 kDa), and ITGB2 (80 kDa). Vinculin (124 kDa) was used as loading control (N = 3). **d** Cell spreading of BJAB WT and BJAB CD151KO cells on ICAM-1 coating was determined using Airyscan confocal microscopy. Representative images are shown, cell area and perimeter are shown in the plots below. Statistical significance was assessed by Mann–Whitney U test (****P < 0.0001). (Magnification 63X, scale bar = 10 µm, N = 3, WT = 146 cells, CD151KO = 148 cells)

Following the identification of the association between CD151 and ITGB2, we analyzed the spatial localization of CD151 I and ITGB2 using confocal microscopy. ITGB2 was correlated with CD151 expression with a Pearson’s coefficient of 0.5 (Fig. 3a). As positive control, anti-CD151 antibody clones 11G5a and 50–6 were used that showed high correlation (Pearson’s coefficient > 0.9) of expression with CD151-GFP in contrast to an isotype control antibody (Pearson’s coefficient 0.2) (Fig. 3a, SFig. 4).

Next, we studied the role of CD151 in ITGB2 function using CD151KO and WT control cells. ITGB2 has been reported to activate pro-survival signals in cancer cells [50]. However, CD151KO and CD151-expressing BJAB and Raji cell lines showed no difference in viability and proliferation (SFig. 5a). We investigated whether CD151 has a role in ITGB2-mediated B cell adhesion. Cell surface expression levels of ITGB2 were comparable between CD151KO and WT BJAB cells, whereas CD151 overexpression resulted in slightly higher levels of ITGB2 (SFig. 6). We determined surface expression of ITGB2-associated alpha chains; namely ITGAL, ITGAM, and ITGAX. BJAB CD151KO cells showed a significant decrease in expression of ITGAL and ITGAM in comparison to BJAB WT cells (Fig. 3b). This was specific for these integrin alpha chains, since expression of a broad panel of other membrane receptors (including known CD151-partners ITGA3 and c-Met) was similar between WT and CD151KO cells (SFig. 5b). Expression of the alpha chains was further validated using western blot with lysates from BJAB WT and CD151KO cells. Total ITGAM protein expression was strongly reduced in BJAB CD151KO cells, while levels of ITGB2 remained the same (Fig. 3c, SFig. 7 for original blots). We then studied the functional consequences by assessing adhesion and cell spreading on ICAM-1 coated surfaces. Whereas B cell adhesion to ICAM-1 was not impaired in CD151KO cells (SFig. 8), we observed clear differences between WT and CD151KO cells in spreading on ICAM-1 coated surfaces by measuring the area and the perimeter of the cells (SFig. 9). BJAB CD151KO were significantly less able to spread on ICAM-1 in comparison to BJAB WT, showing that CD151 is required for ITGB2 function most likely through ITGAM (Fig. 3d).

### B cells express ITGA3-free CD151

**Fig. 4:**
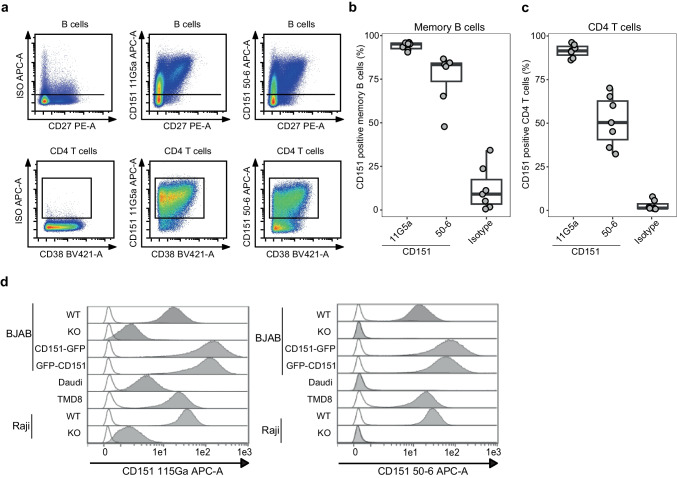
Integrin-free CD151 is the predominant form of CD151 on primary B cells and B cell lymphomas. **a** Representative flow cytometry plots for CD151 stained naive (CD3^−^, CD19^+^, CD27^−^) and memory B (CD3^−^, CD19^+^, CD27^+^) and CD4 T cells (CD3^+^, CD4^+^) from PBMCs (N = 2). Samples were stained with CD151 antibody clones 11G5a or 50–6 and isotype control. For B cells, CD151 expression is plotted against CD27 expression to differentiate between naive and memory B cells. For CD4 T cells, CD151 expression was plotted against CD38. **b** Quantification of percentage of CD151-positive memory B cells. **c** Quantification of percentage of CD151-positive CD4 T cells. **d** B cell lymphoma cell lines with varying CD151 expression (BJAB with over-expressed GFP-tagged CD151) were stained with CD151 antibody clones 11G5a or 50–6 (filled histograms) and isotype control (open histograms)

CD151 not associated with ITGA3 has been reported as “integrin-free” CD151 and shows integrin-independent functions and prognostic value in certain cancers [37]. Therefore, we studied the association of CD151 with ITGA3 in normal healthy B cells and B cell lymphomas. CD151 antibody clone 11G5a was used to detect total CD151 expression (integrin-free and integrin-bound) and antibody clone 50–6 to detect ITGA3 CD151^free^. These studies showed that on B cells, the vast majority of surface CD151 is integrin-free (Fig. 4a). Gated memory B cells that were stained with CD151 clones 11G5a and 50–6 showed identical staining patterns independent of the antibody clone (Fig. 4a,b). This is in contrast to T cells which were all stained with clone 11G5a (CD151^total^) but only a fraction was stained with clone 50–6 (CD151^free^), showing a gradient of expression of integrin-free CD151 (Fig. 4a,c). On a side note, CD38-positive T cells showed the highest levels of integrin-free CD151 (Fig. 4a). Next, integrin-free and total CD151 expression in lymphoma cells was assessed, showing a homogeneous, mostly integrin-free CD151 expression on the surface of different lymphoma cell lines (Fig. 4d), which is in line with primary B cells. Interestingly, CD151 antibody clone 11G5a that is often used in immunohistochemical stainings seemed to bind to an unknown molecule on Raji and BJAB CD151 KO cells (Fig. 4d). This was not observed with CD151 antibody clone 50–6.

### Prognostic power of CD151 and integrin expression in B cell lymphoma

**Fig. 5:**
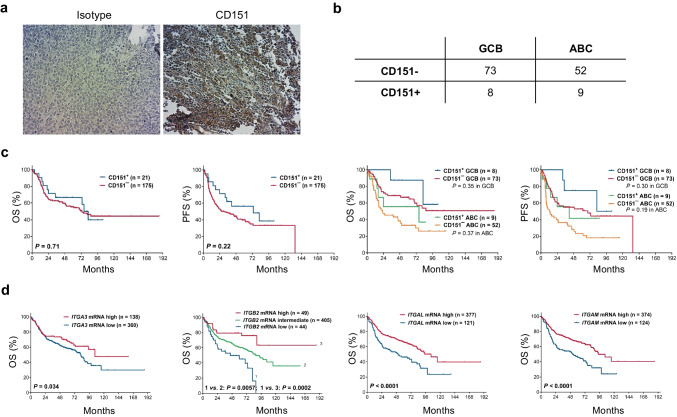
Expression and prognostic effect of CD151 antigen and integrins in patients with DLBCL. **a** Representative CD151 IHC staining with clone E4I9J and isotype control on DLBCL tissues (TMA slides from [44]). **b** Protein expression of CD151 was correlated with ABC DLBCL subtype in CD151 positive samples. **c** Protein expression of CD151 is not a prognostic marker for overall survival (OS) or progression-free survival (PFS) in DLBCL patients (N = 196). **d** OS of CD151 binding partners from IP studies showed integrins as prognostic marker for OS of DLBCL patients: OS was significantly correlated with tissue mRNA expression of ITGA3, ITGB2, ITGAL, and ITGAM. Data was obtained from GSE31312, R-CHOP treated DLBCL (N = 498)

CD151 expression has been well-established to correlate with poor prognosis for patients with different solid cancer types due to enhanced metastasis among other mechanisms, but this has not yet been established for B-cell non-Hodgkin lymphoma [18, 37]. Our study identified higher CD151 expression in lymphomas compared to normal B cells, thus we analyzed CD151 expression levels in relation to clinical outcome in a well-defined cohort of R-CHOP-treated DLBCL patients [44]. Since we found the commonly used CD151 antibody clone 11G5a to be not completely specific for CD151 (Fig. 4d), an alternative CD151 antibody (clone E4I9J) was optimized for immunohistochemistry experiments (Fig. 5a). Only a small proportion (12%) of DLBCL tissues was identified as CD151 positive. Whereas expression of CD151 was predominantly detected in the more aggressive ABC subtype of DLBCL (Fig. 5b), protein expression of CD151 did not correlate with OS or PFS of DLBCL patients independent of the subtype of DLBCL (GCB or ABC) (Fig. 5c). We further investigated clinical outcome of DLBCL patients with respect to expression of identified CD151-interacting proteins. In tissue mRNA expression data, we found significant correlations of OS with ITGA3, ITGB2, and its partners ITGAL and ITGAM expression levels in DLBCL tissues (Fig. 5d).

## Discussion

B cell Non-Hodgkin lymphomas are among the most common type of hematopoietic malignancies. These tumors develop mostly from mature and activated B cells and better understanding of the biological changes of these malignant cells compared to their healthy counterparts may facilitate the development of new therapeutic approaches. Targeting of small four-transmembrane proteins like CD20 and CD37 have been used successfully in the treatment of several B cell cancers [51]. Tetraspanin CD151 has been identified as driver of cancer development and metastasis. Here we report CD151 expression and its molecular interaction with ITGB2 in B cell lymphomas.

Following reports on CD151 expression in T cells, we identified CD151 in B cells where its expression increased upon B cell differentiation. Tetraspanins bind partner proteins with their LEL and often control their surface expression, activation state and/or turnover [7]. Important interaction partners of tetraspanins are integrins [52]. CD151 has been established to strongly interact with laminin-binding integrins (α3β1 and α6β1) and CD151-deficient patients have defects in basement membrane integrity in multiple tissues [14, 53]. The B cells of these patients have not been studied, and defects of murine B cells from CD151-deficient mice have not been reported [42]. Here we identified ITGB2 as novel CD151 partner. ITGB2 forms the heterodimeric lymphocyte function-associated antigen 1 (LFA-1) together with ITGAL and Macrophage-1 antigen (MAC-1) together with ITGAM. LFA-1/ICAM-1 interactions play a crucial role in B cell activation via synapse formation, which is necessary for antigen recognition and activation. LFA-1 regulates B cell activation by lowering the threshold required to form a synapse and is important for B cell adhesion to follicular dendritic cells upon antigen presentation [54, 55]. BJAB lymphoma cells express low levels of ITGAL as partner of ITGB2 in LFA-1, and intermediate levels of ITGAM. The expression of VLA-4 and LFA-1 is increased in memory and marginal zone B cells compared to naïve B cells [56]. Additionally, human memory B cells show greater adhesion capacity to ICAM-1 and VCAM-1. Another study reported integrin beta 7 (ITGB7) as potential CD151 interaction partner [39]. Our immunoprecipitation data also revealed the presence of ITGB7, suggesting an interaction between this molecule and CD151.

Cell spreading on ICAM-1 was significantly affected upon CD151KO, which is likely caused by decreased expression of αMβ2 and αLβ2 integrin complexes. This could result in defective outside-in signaling in line with previous studies on CD151 in platelets [57], and further cytoskeleton reorganization is required for integrin dependent cell spreading. It is possible that CD151 is also required for de-adhesion under flow conditions which has been shown for tetraspanin CD81 that was found to stimulate VLA-4-mediated leukocyte rolling and arrest on VCAM-1 under shear flow [58]. While investigating CD151 expression, we found that the broadly used clone 11G5a has affinity for an off-target protein. This was most prominent in intracellular flow cytometry and IHC stainings and should be taken into account for future studies. Using multiple antibodies, CD151 expression was highest on activated B cells, including memory and plasma cells. This expression pattern of CD151 may indicate a role for CD151 in secondary lymphoid organs such as increased cell–cell adhesion in the germinal centers. Alternatively, CD151 could be required for de-adhesion of cells that need to be motile and leave the secondary lymph nodes. Lastly, CD151 has been found in the immunological synapse of T cells. Its depletion decreased the re-localization of α4β1 integrin to the immune synapse in T cells together with decreased FAK phosphorylation [59].

CD151 was recently reported as T cell activation marker based on a population of activated proliferating T cells with high levels of CD151 but absence of activation markers like CD25 and CD38 [60]. Here, CD151 antibodies that solely detect integrin-free CD151 were used. Our data shows that CD151 is homogeneously expressed on T cells but that integrin-free CD151 is only found on a fraction of T cells. Further, T cells with high levels of integrin free-CD151 expressed more CD38. Overall, this proposes CD151 to not be a classical activation marker, but T cell activation seems to be marked by the integrin association status of CD151.

With extensive studies in malignant and non-malignant cells, CD151 has emerged as important protein in regulating cell motility and metastasis formation, whereby increased CD151 expression has been associated with a more aggressive clinical course and poor prognosis [8, 9, 15, 18–26]. Although not directly related to clinical outcome of patients with DLBCL, CD151 was predominantly detected in the more activated (ABC) DLBCL subtype. Tetraspanins can have multiple binding partners that they coordinate, and as such their expression may predict opposite outcomes of survival between different cancer types [61]. The functions of CD151 in cell motility have been linked to the integrin association status of CD151 [8, 9]. Investigating CD151 integrin association in different cancer types could improve prognostic power for survival analysis. In this study we report increased expression of CD151 in lymphomas compared to healthy B cells. We identified a new association of CD151 with ITGB2 in lymphoma cells that is important for cell spreading. Contrary to T cells, B cells, and B cell lymphomas expressed CD151 with a free ITGA3 binding domain, and targeting integrin-free CD151 in DLBCL may represent a new target for immunotherapy.

## Supplementary Information

Below is the link to the electronic supplementary material.

Supplementary file1 (PDF 57506 KB)

Supplementary file2 (DOCX 24 KB)

Supplementary file3 (XLSX 63 KB)

## Data Availability

All data are included within the article.
