# Supplementary material for: CD151 interacts with integrin beta 2 in B cell lymphomas
Source: Cell Mol Life Sci. 2025 Jun 4;82(1):221. doi: 10.1007/s00018-025-05747-0 (PMC12137850; doi:10.1007/s00018-025-05747-0)
Supplement: Supplementary file 1 — Supplementary file1 (PDF 57506 KB) [file 18_2025_5747_MOESM1_ESM.pdf]

CD151 interacts with integrin beta 2 in B cell lymphomas

Philipp M. Hagemann[1], Angelique N. Kenyon[1], Alfredo Cabrera-Orefice[2], Abbey B. Arp[1], Eva A. M. Hesius[3], Michiel van den Brand[4,5], Sjoerd J. van Deventer[1], Daphne de Jong[6], Blanca Scheijen[5], Zijun Y. Xu-Monette[7], Ulrich Brandt[2], Cornelia G. Spruijt[8], Michiel Vermeulen[8,9], Martin ter Beest[1], Ken H. Young[7], Annemiek B. van Spriel[1,*]

[1]Department of Medical BioSciences, Radboud Institute for Medical Innovation, Radboud University Medical Center, Nijmegen, The Netherlands.
[2]Radboud Institute for Medical Innovation, Radboud University Medical Center, Nijmegen, The Netherlands.
[3]Department of Hematology, Radboud University Medical Center, Nijmegen, The Netherlands.
[4]Pathology-DNA, Rijnstate Hospital, Arnhem, The Netherlands.
[5]Department of Pathology, Radboud University Medical Center, Nijmegen, The Netherlands
[6]Department of Pathology, Antoni van Leeuwenhoek Hospital/The Netherlands Cancer Institute, Amsterdam, The Netherlands.
[7]Hematopathology Division and Department of Pathology, Duke University Medical Center, Durham, NC, USA.
[8]Department of Molecular Biology, Faculty of Science, Oncode Institute, Radboud University Nijmegen, Nijmegen, The Netherlands.
[9]Division of Molecular Genetics, The Netherlands Cancer Institute, Amsterdam, The Netherlands.

*Correspondence: annemiek.vanspriel@radboudumc.nl

SFig. 1

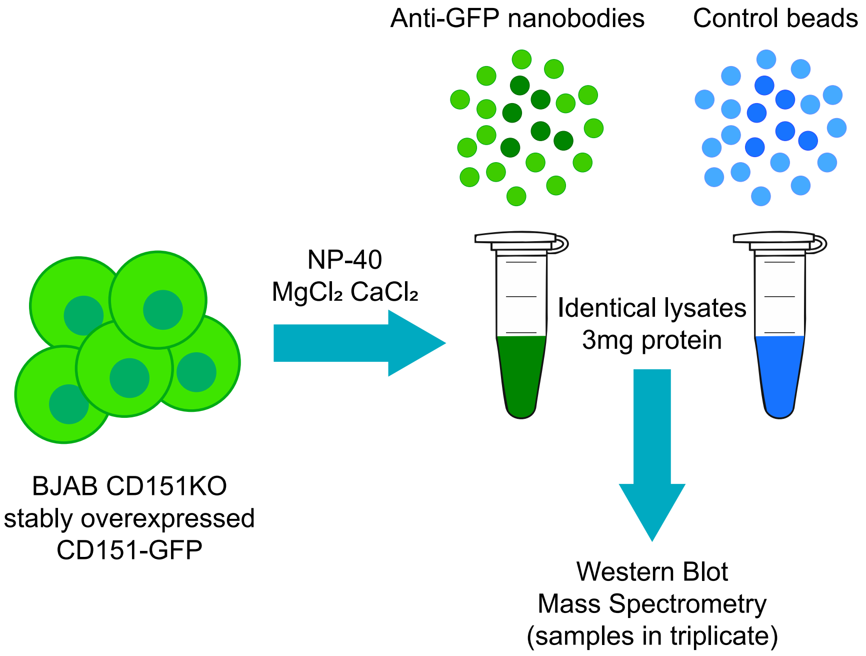

SFig 1. Schematic representation of GFP pulldown setup. This system allows for studying protein interactions mediated by the LEL of CD151, which is not disturbed by the GFP pulldown. Lymphoma cells were lysed in IGEPAL CA-630 and lysates were split and incubated with either affinity (GFP nanobody) beads or control beads.

SFig. 2

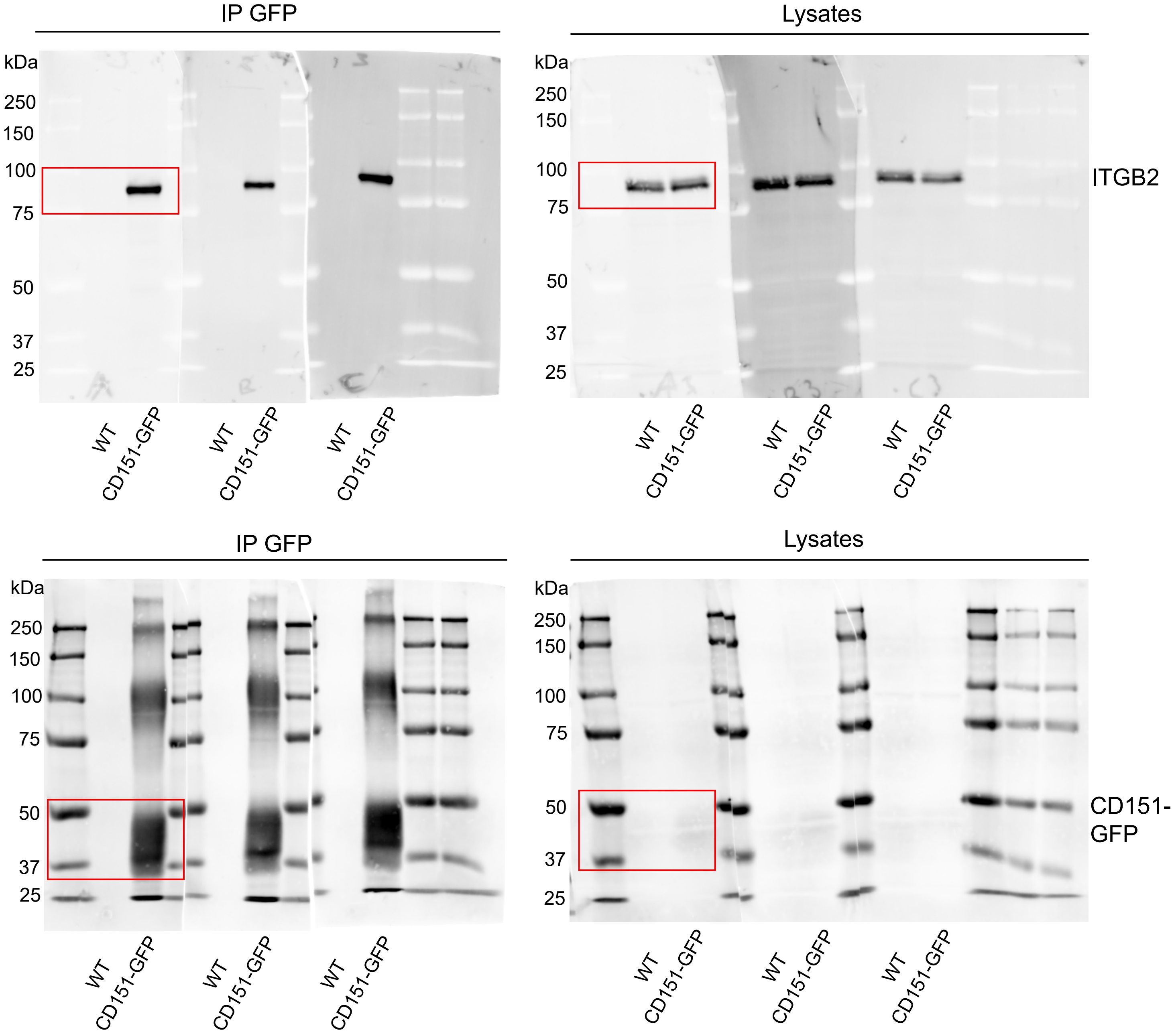

SFig 2. Original western blots corresponding to Fig. 2d (N=3).

SFig. 3

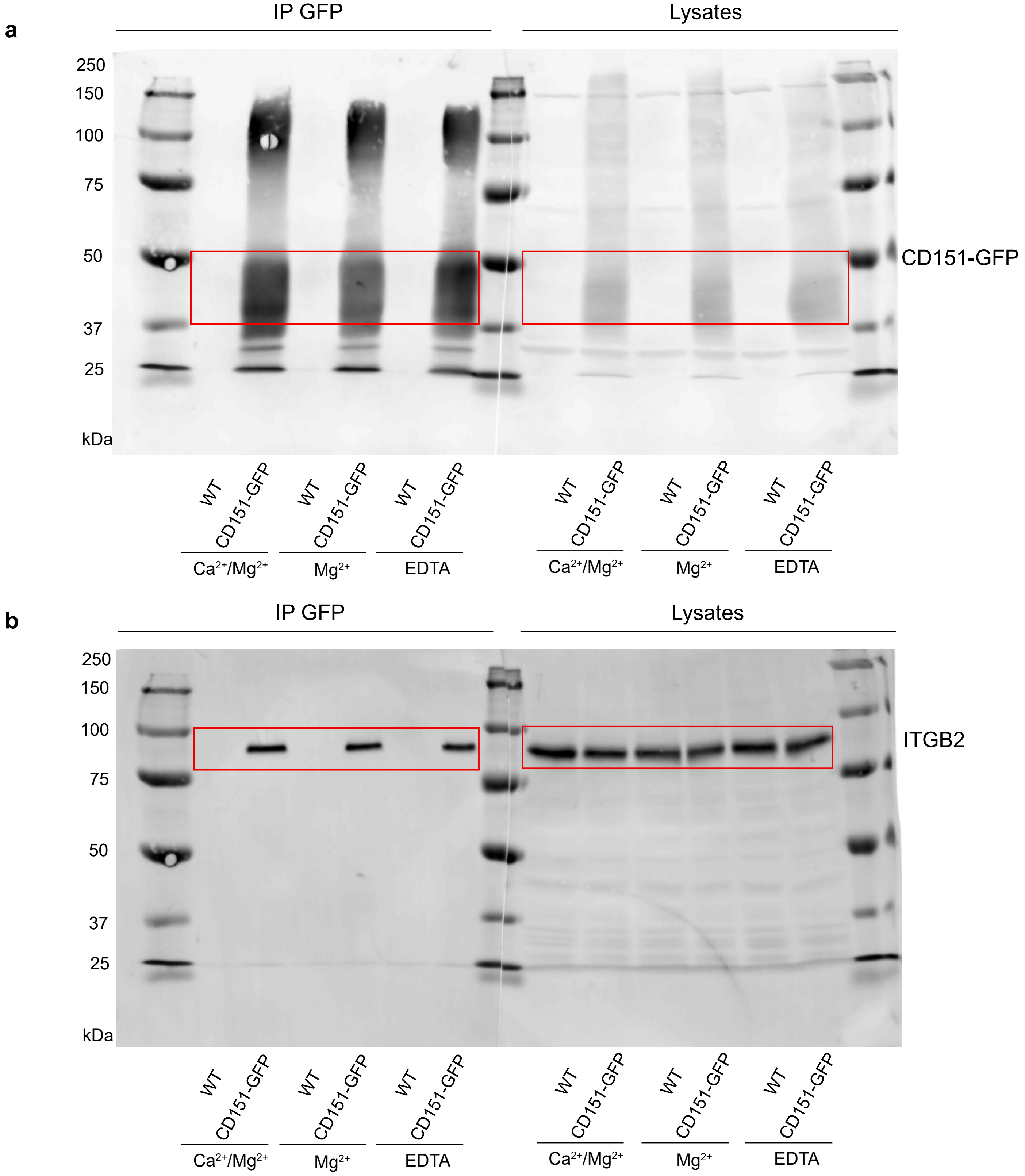

SFig 3. Interaction of CD151 with ITGB2 is independent of divalent cations. Pulldown of CD151 by GFP-trap beads in lysates from BJAB WT and BJAB CD151-GFP. (**a**) Western blot stained for anti-GFP. (**b**) Western blot stained for anti-ITGB2.

SFig. 4

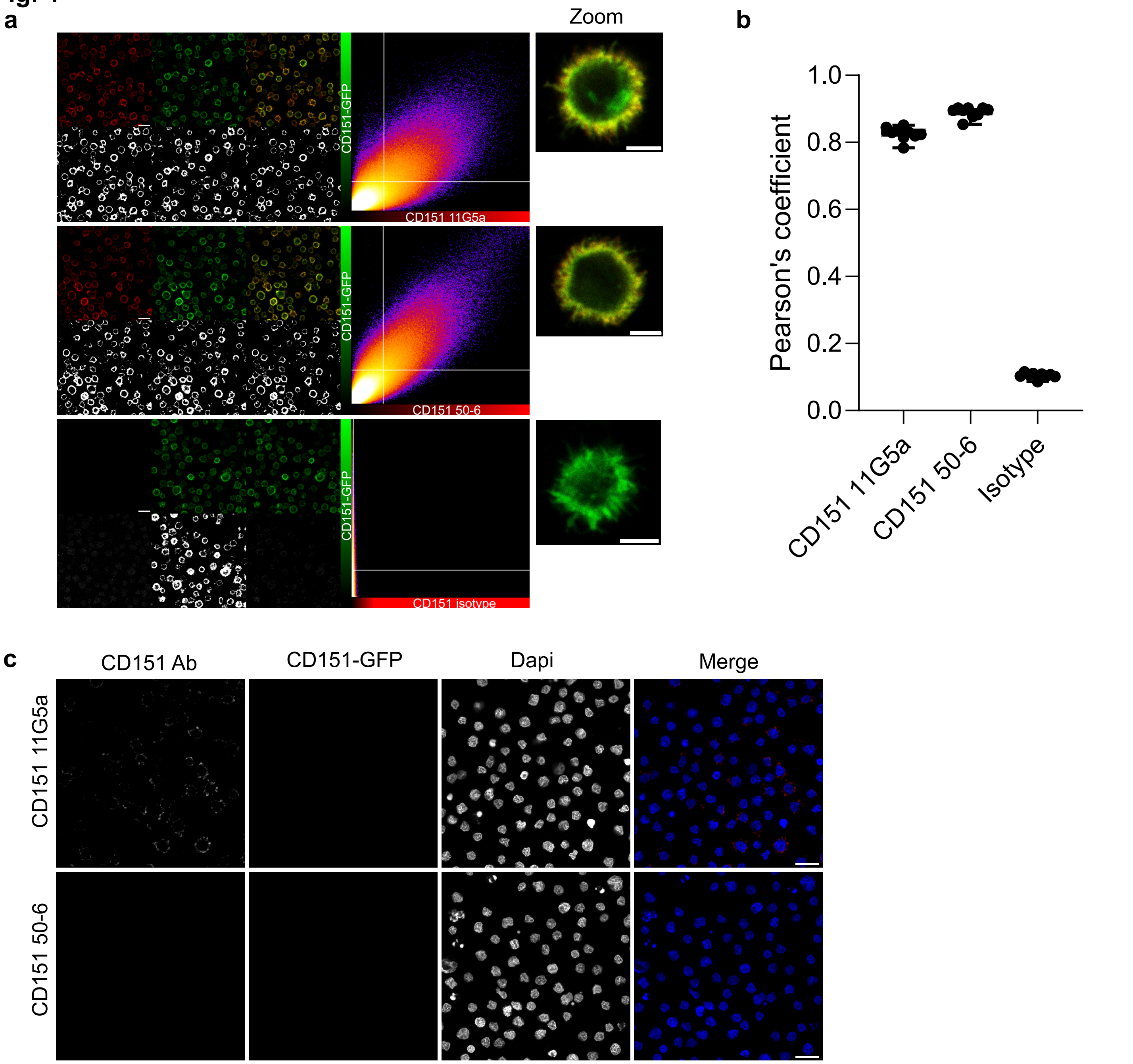

SFig 4. Colocalization of CD151-GFP overexpression with CD151 antibody clones. (**a**) Representative fluorograms of CD151 colocalization with anti-CD151 11G5a, anti-CD151 50-6, and isotype (negative control). BIOP-JACoP was used for determination of colocalization. (**b**) Pearson's coefficients of the analyzed images are shown in the plot. (N=2, 4 images per experiment). (**c**) CD151KO cells stained for anti-CD151 11G5a and anti-CD151 50-6 (N=2). (Magnification 40X, scale bar = 20 μm or 5 μm for zoom)

SFig. 5

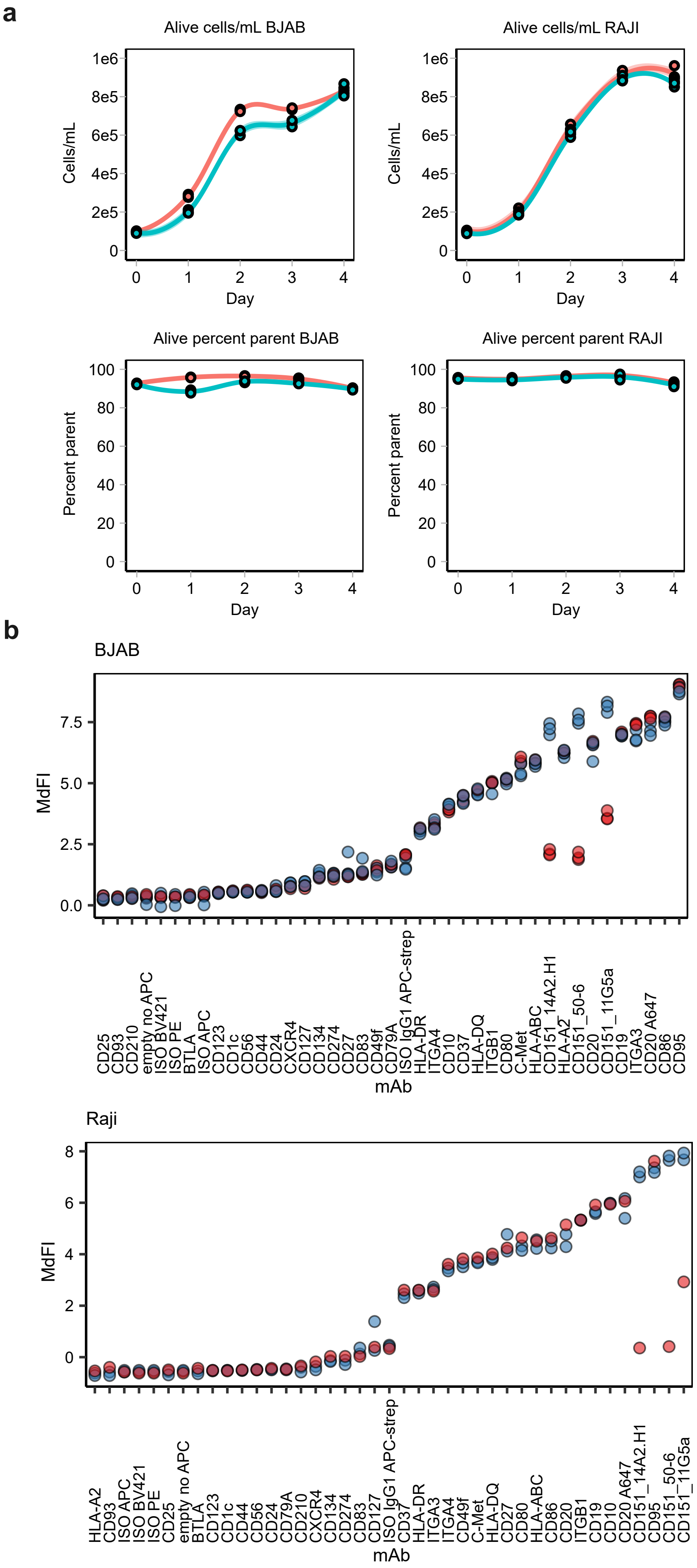

SFig 5. (**a**) Proliferation and viability of BJAB and Raji cell lines (blue) compared to CD151KO cell lines (red). (**b**) Expression of B cell surface markers on BJAB and Raji cell lines from CD151KO cells (red) compared to age matched transfection controls (blue). Overlapping expression is represented in purple.

SFig. 6

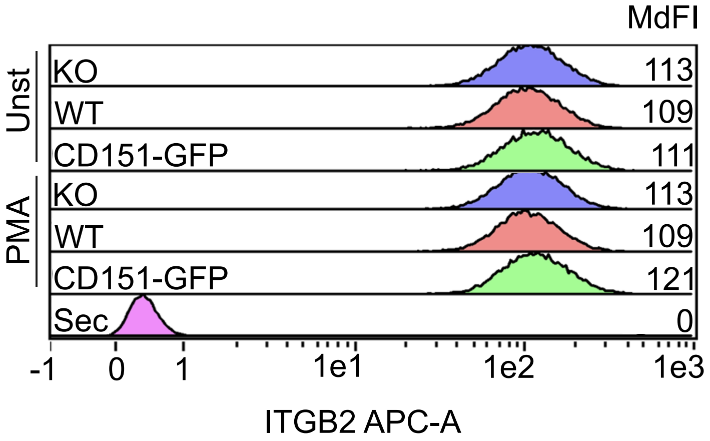

SFig 6. Surface expression of ITGB2 of BJAB cell lines expressing different amounts of CD151 was measured by flow cytometry. Histograms for ITGB2 staining on unstimulated and PMA-treated cell lines and for secondary staining alone are shown.

SFig. 7

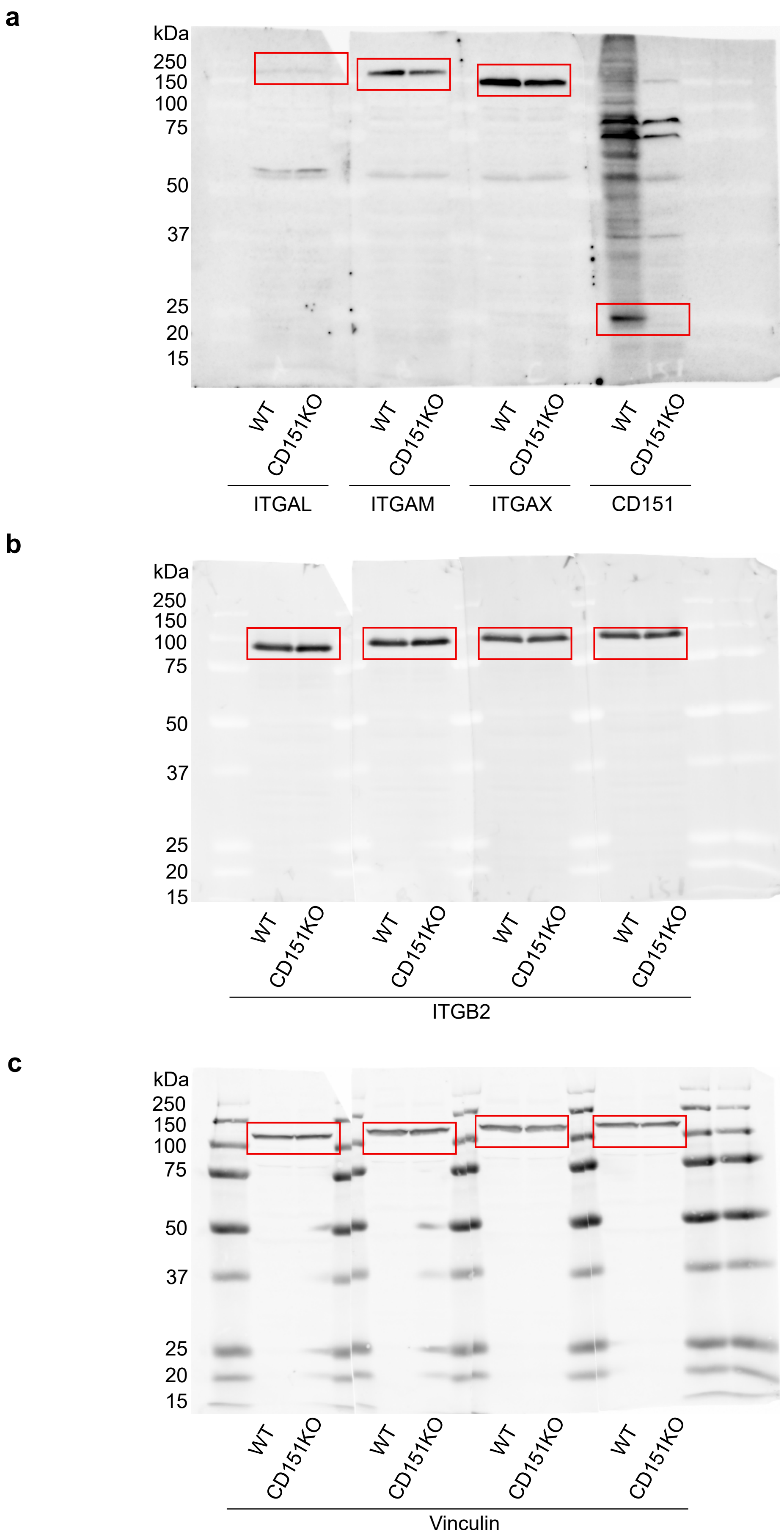

SFig 7. Original western blots corresponding to Fig. 3c. (**a**) Western blot stained for ITGAL, ITGAM, ITGAX and CD151. (**b**) Western blot stained for ITGB2. (**c**) Western blot stained with vinculin as loading control. (N=3, red boxes indicate integrin bands)

SFig. 8

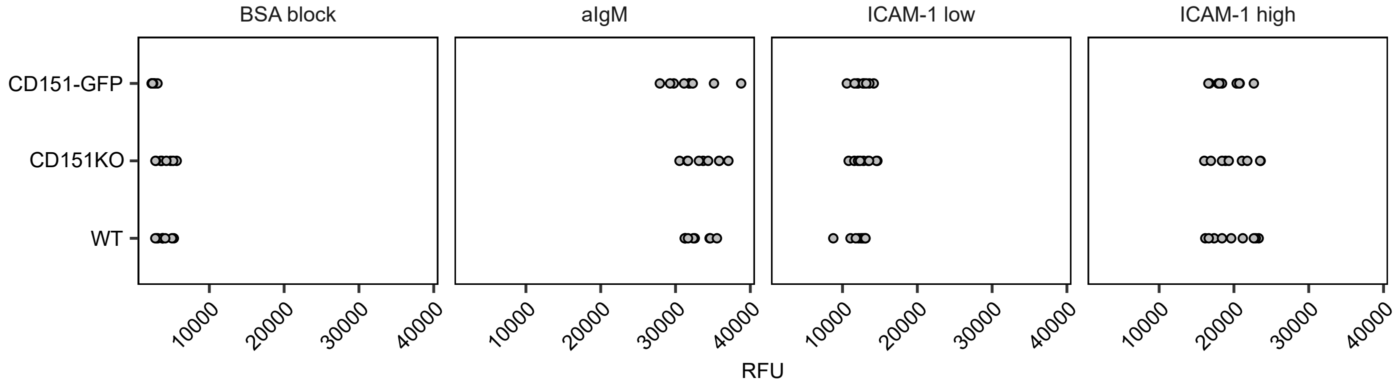

SFig 8. Adhesion of BJAB cell lines to integrin beta 2-ligand ICAM-1 with low (1 µg/mL) and high (10 µg/mL) density compared to anti-IgM coating (positive control) and BSA coating (negative control) RFU= Relative fluorescence units. (N = 3)

SFig. 9

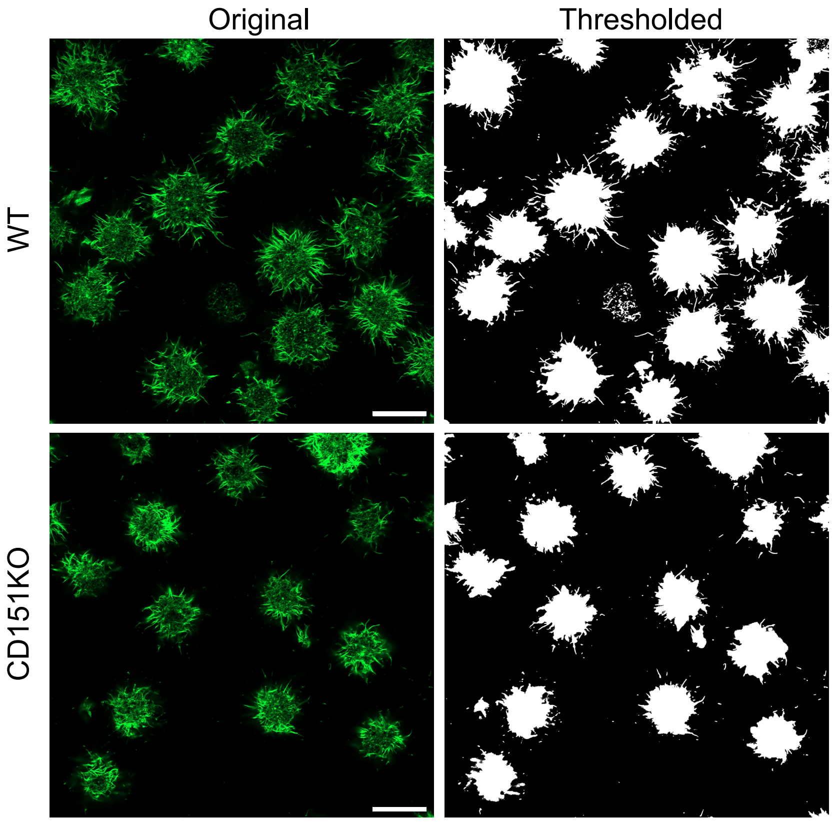

SFig 9. Quantification of cell spreading using actin for membrane dye. Images were taken on the cell/-glass interface (left panel). Images were thresholded (right panel), followed by measuring the cell area and perimeter. The area represents the amount of space inside the cell boundary while the perimeter represents the total length of the cell boundary. Using FIJI the cell was traced in the thresholded image, added to the roi manager, followed by determination of the area and perimeter using the measurement tool. (Magnification 63X, scale bar = 10 μm)
