# Supplementary material for: CD151 interacts with integrin beta 2 in B cell lymphomas
Source: Cell Mol Life Sci. 2025 Jun 4;82(1):221. doi: 10.1007/s00018-025-05747-0 (PMC12137850; doi:10.1007/s00018-025-05747-0)
Supplement: Supplementary file 2 — Supplementary file2 (DOCX 24 KB) [file 18_2025_5747_MOESM2_ESM.docx]

**CD151 interacts with integrin beta 2 in B cell lymphomas**

Philipp M. Hagemann^1^, Angelique N. Kenyon^1^, Alfredo Cabrera-Orefice^2^, Abbey B. Arp^1^, Eva A. M. Hesius^3^, Michiel van den Brand^4,5^, Sjoerd J. van Deventer^1^,Daphne de Jong^6^, Blanca Scheijen^5^, Zijun Y. Xu-Monette^7^, Ulrich Brandt^2^, Cornelia G. Spruijt^8^, Michiel Vermeulen^8,9^, Martin ter Beest^1^, Ken H. Young^7^, Annemiek B. van Spriel^1,*^

^1^Department of Medical BioSciences, Radboud Institute for Medical Innovation, Radboud University Medical Center, Nijmegen, The Netherlands.

^2^Radboud Institute for Medical Innovation, Radboud University Medical Center, Nijmegen, The Netherlands.

^3^Department of Hematology, Radboud University Medical Center, Nijmegen, The Netherlands.

^4^Pathology-DNA, Rijnstate Hospital, Arnhem, The Netherlands.

^5^Department of Pathology, Radboud University Medical Center, Nijmegen, The Netherlands

^6^Department of Pathology, Antoni van Leeuwenhoek Hospital/The Netherlands Cancer Institute, Amsterdam, The Netherlands.

^7^Hematopathology Division and Department of Pathology, Duke University Medical Center, Durham, NC, USA.

^8^Department of Molecular Biology, Faculty of Science, Oncode Institute, Radboud University Nijmegen, Nijmegen, The Netherlands.

^9^Division of Molecular Genetics, The Netherlands Cancer Institute, Amsterdam, The Netherlands.

* Correspondence: annemiek.vanspriel@radboudumc.nl

**Supplementary information**

**Table S1:** Antibodies and dilutions

| **Antibody**  **Target** | **Clone** | **Host species** | **Target species** | **Supplier/#** | **Method** | **Concentration / Dilution** |
| --- | --- | --- | --- | --- | --- | --- |
| Anti-Human IgM | Goat F(ab')2 | Goat | Human | SouthernBiotech  #2022-01 | IF | 10 µg/mL |
| ICAM-1-FC | Recombinant | Chimera | Human | Biolegend  #552906 | IF | 10 µg/mL |
| CD151 | 11G5a | Mouse | Human | BioRad #MCA1856 | IF,FC,WB | 5 µg/mL  10 µg/mL |
| CD151 | 50-6 | Mouse | Human | Biolegend #350402 | IF,FC,WB | 5 µg/mL |
| CD151 | 50-6 | Mouse | Human | Sigma  #SAB4700796 | IF | 10 µg/mL |
| Mouse isotype IgG1-APC | MOPC-21 | Mouse | Human | Biolegend #981802 | IF,FC,WB | 5 µg/mL |
| Mouse isotype IgG1 | MOPC-21 | Mouse | Human | Biolegend  #400102 | IF | 10 µg/mL |
| CD151 (E4I9J) XP®Rabbit mAb | E4I9J | Rabbit | Human | Cell Signaling #96282 | IHC,WB | 1:400  1:1000 |
| Rabbit isotype IgG | Rabbit IgG | Rabbit | Human | Jackson IE #AB_2337118 | IHC | 10 µg/mL |
| Phalloidin-AF488 | - | - | - | ThermoFisher Scientific  #A12379 | IF | 1:50 |
| CD27 PE | M-T271 | Mouse | Human | Biolegend  # 986908 | FC | 1:50 |
| CD19 FITC | HIB19 | Mouse | Human | Biolegend  # 302256 | FC | 1:50 |
| CD3 PE-Cy7 | HIT3a | Mouse | Human | Biolegend  # 300316 | FC | 1:50 |
| CD38 BV421 | HB-7 | Mouse | Human | Biolegend  # 356618 | FC | 1:50 |
| CD18 | Ts1/18 | Mouse | Human | Biolegend  # 302102 | FC | 1:50 |
| ITGA6 | GoH3 | Rat | Human  /Mouse | Biolegend  # 313602 | FC | 1:50 |
| ITGAL | E5S9K | Rabbit | Human | Cell Signaling Technologies  #26703 | WB | 1:1000 |
| ITGAL-APC | MEM-25 | Mouse | Human | Immunotools  #21270116S | FC | 1:20 |
| Mouse isotype IgG1-APC | PPV-06 | Mouse | Human | Immunotools  #21275516S | FC | 1:20 |
| ITGB1 | TS2/16 | Mouse | Human | Biolegend  # 303002 | FC | 1:50 |
| ITGAM | D6X1N | Rabbit | Human | Cell Signaling Technologies  #49420 | WB | 1:1000 |
| ITGAM-APC | Bear1 | Mouse | Human | Beckman Coulter  #A87782 | FC | 1:5 |
| ITGAX | D3V1E | Rabbit | Human | Cell Signaling Technologies  #45581 | WB | 1:1000 |
| ITGAX-APC | B-ly6 | Mouse | Human | BD #559877 | FC | 1:5 |
| Mouse isotype IgG-APC | P3.6.2.8.1 | Mouse | Human | eBioscience  #174714-82 | FC | 1:10  1:100 |
| CD49c ITGA3 | 17C6 | Mouse | Human | BioRad  # MCA1948 | FC | 1:50 |
| Vinculin | hVIN-1 | Mouse | - | Sigma-Aldrich  # V9131 | WB | 1:1000 |
| α-ALFA-AF647 | 1G5 | - | - | NanoTag Biotechnologies #N1502-AF647-L | WB | 1:2000 |
| Mouse anti-GFP | 7.1/13.a | Mouse | - | Roche  #11814460001 | WB | 1:2000 |
| Rabbit-anti‐GFP | Polyclonal | Rabbit | - | Rockland #600‐401‐215L | WB | 1:1000 |
| Goat anti-rabbit-HRP | Polyclonal | Goat | Rabbit | Jackson ImmunoResearch  #111-035-003 | WB | 1:10000 |
| Goat‐anti rabbit IRDye800 | Polyclonal | Goat | Rabbit | Li-Cor  #926‐32211 | WB | 1:5000 |
| Donkey-anti rabbit IRDye800 | Polyclonal | Donkey | Rabbit | Li-Cor  #926-32213 | WB | 1:5000 |
| Donkey-anti mouse IRDye680 | Polyclonal | Donkey | Mouse | Li-Cor  #926-68022 | WB | 1:5000 |

IF: Immunofluorescence, FC: Flow cytometry, WB: Western blotting

**Table S2:** Primers and gRNA sequences

| ITGB2_1 | agaagactgaccgagtgagcggccgcGGCGGACCGGTCGCCACC |
| --- | --- |
| ITGB2_2 | tctcagctcctcctccagtctgctgggACTCTCAGCAAACTTGGGGTTCATGACCG |
| Guide RNA sequences for CD151 (fwd, rev) | \| CACCGAGCAATTGTAGGTAAACAGC \| \| --- \| \| AAACGCTGTTTACCTACAATTGCTC \| |

**Table S3:** Mass spectrometry quantification of immunoprecipitation of CD151-GFP compared to non-capturing bead control. See excel file.
